# Synthesis and cloning of long repeat sequences using single-stranded circular DNA

**DOI:** 10.3389/fbioe.2023.1115159

**Published:** 2023-03-09

**Authors:** Afsana Bhuiyan, Shuichi Asakawa

**Affiliations:** Laboratory of Aquatic Molecular Biology and Biotechnology, Graduate School of Agricultural and Life Sciences, The University of Tokyo, Tokyo, Japan

**Keywords:** repeat sequence, cell-free synthetic biology, in vitro cloning, neurodegenerative diseasaes, spinocerebellar ataxia (SCA) 31, benign adult familial myoclonic epilepsy (BAFME)

## Abstract

Non-coding repeat expansion causes several neurodegenerative diseases, such as fragile X syndrome, amyotrophic lateral sclerosis/frontotemporal dementia, and spinocerebellar ataxia (SCA31). Such repetitive sequences must be investigated to understand disease mechanisms and prevent them, using novel approaches. However, synthesizing repeat sequences from synthetic oligonucleotides is challenging as they are unstable, lack unique sequences, and exhibit propensity to make secondary structures. Synthesizing long repeat sequence using polymerase chain reaction is often difficult due to lack of unique sequence. Here, we employed a rolling circle amplification technique to obtain seamless long repeat sequences using tiny synthetic single-stranded circular DNA as template. We obtained 2.5–3 kbp uninterrupted TGGAA repeats, which is observed in SCA31, and confirmed it using restriction digestion, Sanger and Nanopore sequencing. This cell-free, *in vitro* cloning method may be applicable for other repeat expansion diseases and be used to produce animal and cell culture models to study repeat expansion diseases *in vivo* and *in vitro*.

## Introduction

The discovery that simple tandem repeats or microsatellites can cause neurological diseases was revolutionary in the field of neurodegenerative disorders. Nearly 50 neurological diseases have been identified so far, of which 26 diseases are related to the repeat expansion in coding, non-coding, intron and 5′and 3′UTR regions (Rohilla and Gagnon, 2017; Paulson, 2018; Chintalaphani et al., 2021). Among the repeat expansion-related diseases, spinocerebellar Ataxia type 31 (SCA31) is caused by the repeat expansion of 2.5 to 3.8 kbp pentanucleotide TGGAA, where a pure (TGGAA)_n_ extended for at least 110 repeats in the intron region of the BEAN gene (Sato et al., 2009), whereas benign adult familial myoclonic epilepsy (BAFME1) is associated with the repeat expansion of 105 to 3,680 units of TTTCA (Cen et al., 2018; Ishiura et al., 2018). Such diseases can be classified based on the DNA sequences of repeat units (trinucleotide, tetranucleotide, pentanucleotide, or hexanucleotide). To develop transgenic models of these diseases, we need to obtain disease sequences from patients, except for some special cases (Mizielinska et al., 2014; Swinnen et al., 2018). However, this is often difficult due to ethical regulations and unavailability of patient-derived genomic DNA. In addition, the sizes of disease sequences are limited to that of the patients. Yet, longer DNAs are desirable for developing disease models because of the anticipation found in the repeat diseases (Wells, 1996). In the case of non-repeat mutation disorders, disease genes can be synthesized and studied without patient sources. Moreover, methods to obtain uninterrupted long repeats are lacking owing to the technical obstacles associated with amplification and cloning. Recombinant vectors containing these synthetic repeats can have numerous applications in biological, medical, and bioengineering research. These vectors can be used for studying repeat associated-non-AUG translation (RAN polypeptides) as well as formation of RNA foci and its interaction with RNA binding proteins (Lee et al., 2013; Malik et al., 2021).

Several methods have been described for trinucleotide repeat synthesis using synthetic oligonucleotides. For example, 20 bp trinucleotide repeat has been used as template as well as primer for conventional polymerase chain reaction (PCR) and cloned in a vector (Ordway and Detloff, 1996). Synthesis of long iterative polynucleotide (SLIP) and non-template PCR method for trinucleotide repeat synthesis was developed based on the theory that filling gaps leads to repeat expansion (Takahashi et al., 1999). Another ligation-based method required iterative ligation reactions to obtain expanded repeats (Kim et al., 2005). Alternatively, concatenated DNA was obtained by random insertion of restriction sites using ligation and PCR (Jiang et al., 1996). PCR has also been used to obtain repeat sequences from DNA sources for polyglutamine expansions disease like Huntington’s disease and spinocerebellar ataxia type 10 (Laccone et al., 1999; Peters and Ross, 1999; Michalik et al., 2001; Matsuura and Ashizawa, 2002). Amplification of dimerized expanded repeats (ADER) method was developed to obtain 2,000 CTG repeats using phi29 DNA polymerase in a cell free system (Osborne and Thornton, 2008). In rolling circle amplification or RCA (also known as hyperbranched amplification), small single stranded circular DNA is used as a template for amplification (Fire and Xu, 1995). This method has a low error rate, strong strand displacement activity, high processivity, and uses circular DNA for isothermal amplification (Hafner et al., 2001). Here we describe a cell-free synthetic method taking SCA31 and BAFME1 as examples for the synthesis and cloning of long repeat sequences using rolling circle amplification. As we were able to reproducibly obtain long repeat sequences in a cell-free manner, we regarded this method as a kind of “*in vitro* cloning”.

## Material and methods

Table 1 shows all oligonucleotide sequences used in the experiment. Repeat single-stranded DNA (ssDNA) with phosphorylated 5′ end (80 nt, (TGGAA)_16_, HPLC purified) was obtained from Shanghai Generay Biotech Co., Ltd. (Generay, Shanghai, China). For RCA control, M13 DNA (90 nt) without repeat sequence, was obtained from Hokkaido System Science Co., Ltd. (Hokkaido, Japan). The 20 pmol ssDNA was incubated overnight at 60°C with CircLigase II (Lucigen CL9021K) according to the manual condition to obtain single-stranded circular DNA. The ligase was inactivated by heating at 80°C for 10 min. To remove excess ssDNA, exonuclease T (NEB M0265) was used at 25°C for 30 min and inactivated at 65°C for 20 min. Alternatively Exonuclease I (NEB M0293) can be used (See the results & discussion). This clean circular DNA (2 pmol) was used as a template for RCA, which was performed using Bst DNA polymerase, large fragment (NEB M0275) with 5′phospholated forward and reverse primers (Sca31_RCA_F, Sca31_RCA_R) at 60°C for 12 h. The amplified products were treated with 5 U mung bean nuclease (Takara Bio, 2420) and 0.5 U nuclease P1 (Wako 145-08221) at 37°C for 10 min. After digestion, the DNA was run on a 1.5% agarose gel and 2-3 kbp DNA samples were excised from the gel and purified with the Fastgene gel/PCR extraction kit (Nippon genetics co. FG-91302). The pIRES2 DsRed-Express2 vector (Takara Bio, 632540) was digested by AfeI (NEB R0652) and Shrimp Alkaline Phosphatase (rSAP) (NEB M0371) to prepare blunt ended vector. pIRES and the purified 2-3 kbp DNA insert were ligated for 2 h using T4 DNA Ligase (NEB M0202) and electroporated in Stable Competent *E. coli* (NEB C3040) cells. After 30 min recovery of competent cells, they were plated on kanamycin-supplemented culture plates and incubated at 30°C overnight. The insert lengths of selected clones were checked by PCR amplification using Phusion High-Fidelity DNA Polymerase (NEB M0530) and vector primers (pIRES_F pIRES_R) and by restriction digestion with NheI-HF & EcoRI-HF whose sites are located close to the cloning site, and by Sanger sequencing. The same process was applied to another repeat TTTCA (BAFME1) (80 nt, (TTTCA)_16_, HPLC purified) to check the versatility of the method. The whole length of one SCA31 plasmid was sequenced using Oxford Nanopore Technology (ONT) (Stevanovski et al., 2022). Library preparation with the ligation sequencing kit (SQK-LSK110) was followed by sequencing on Flongle Flow Cell (FLO-FLG001). Multiple alignment using fast fourier transform (MAFFT) on raw sequence data and consensus sequence polishing by the Medaka tool (Lee et al., 2021) were employed to construct a complete plasmid map.

**TABLE 1:** Sequences for oligonucleotides used in ssDNA template preparation, rolling circle amplification, PCR amplification check and Sanger sequencing.

Oligonucleotides	Sequence (5’−3′)
80nt TGGAA ssDNA	TGG​AAT​GGA​ATG​GAA​TGG​AAT​GGA​ATG​GAA​TGG​AAT​GGA​ATG​GAA TGG​AAT​GGA​ATG​GAA​TGG​AAT​GGA​ATG​GAA​TGG​AA
Sca31_RCA_F	TGGAATGGAATGGAA
Sca31_RCA_R	TTCCATTCCATTCCA
pIRES_F	GTA​ACA​ACT​CCG​CCC​CAT​T
pIRES_R	GGT​ACC​GTC​GAC​TGC​AGA​A
M13_90mer ssDNA (Control for RCA)	GTA​AAA​CGA​CGG​CCA​GTA​AAC​AGT​GAC​CAT​GAT​AGT​GGC​CAC​CCT​GCA​ACC​GTG​TTG​TTT​GTC​AGG​TTC​ATT​TGT​CAT​AGC​TGT​TTC​CTG
M13_RCA_F	CTGCAACCGTGTTGTTT
M13_RCA_R	GGTGGCCACTATCAT
80 nt TTTCA ssDNA	TTT​CAT​TTC​ATT​TCA​TTT​CAT​TTC​ATT​TCA​TTT​CAT​TTC​ATT​TCA​TTT​C ATT​TCA​TTT​CAT​TTC​ATT​TCA​TTT​CAT​TTC​A
BAFME1_RCA_F	TTTCATTTCATTTCA
BAFME1_RCA_R	TGAAATGAAATGAAA

## Results & discussion

**FIGURE 1:**
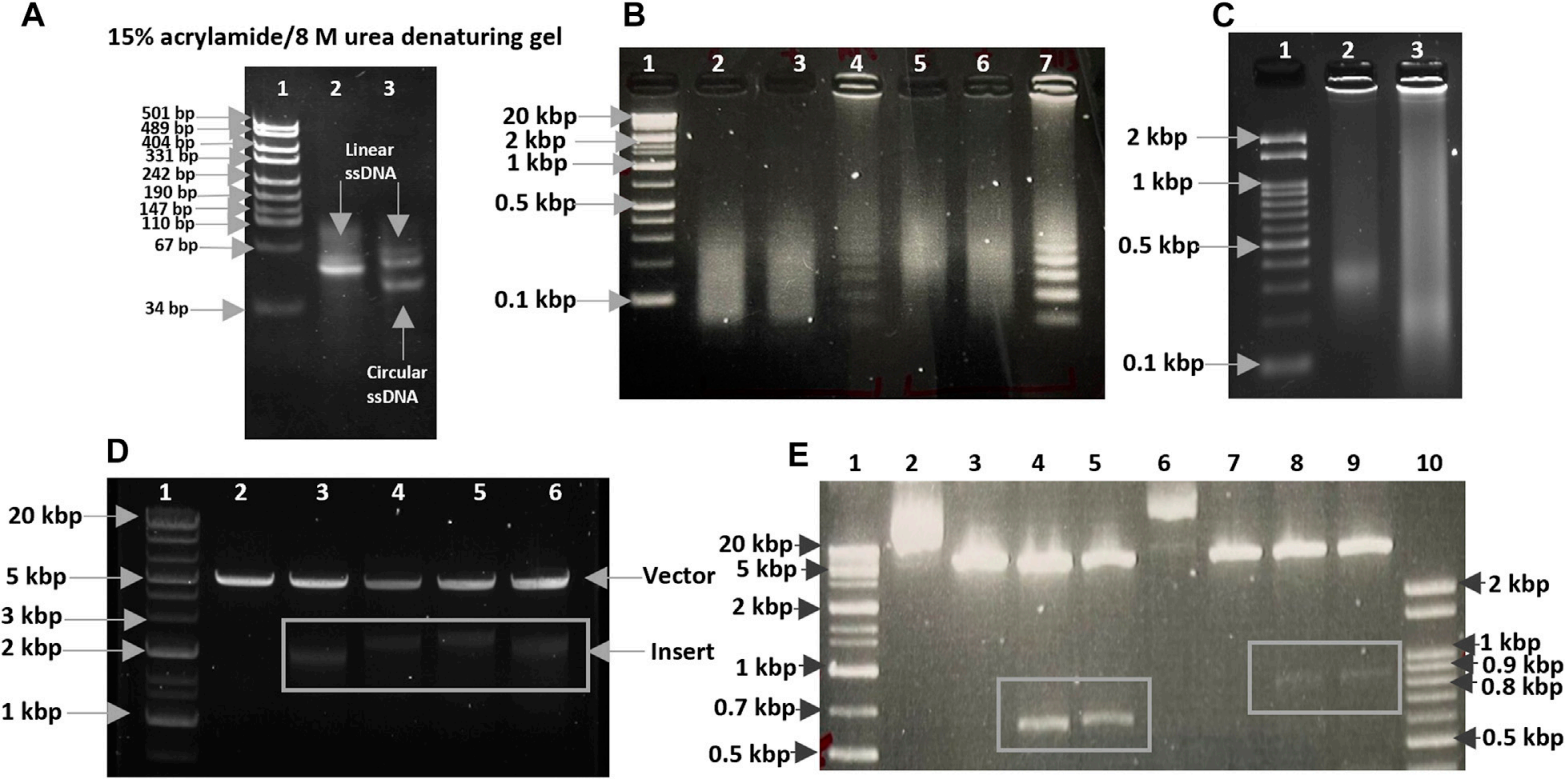
**(A)** CircLigase II ssDNA ligase converted 80 nt linear ssDNA oligos into circular ssDNA. Lane 1. pUC19 DNA/MspI (HpaII) Marker (Thermo Scientific); Lane 2. 80 nt linear ssDNA oligos (upper band); Lane 3. Unreacted 80 nt linear ssDNA oligos (upper band), and 80 nt circular ssDNA product (lower band). **(B)** RCA products after 1 h incubation. Lane 1. Size maker, Gene Ladder Wide 1 (0.1–20 kbp) (Nippon genetics co. 313-06961); Lane 2,3,5,6. RCA for SCA31; Lane 4,7. RCA for M13_90mer. **(C)** RCA products after 12 h incubation. Lane 1. Size maker, Gene Ladder 100 (0.1-2 kbp) (Nippon genetics co. 316-06951); Lane 2. RCA for SCA31 (12 h); Lane 3. RCA digested with 5 U mung bean nuclease and 0.5 U nuclease P1. **(D)** Size analysis of the TGGAA plasmids. Lane 1. Size maker, Gene Ladder Wide 1 (0.1–20 kbp) (Nippon genetics co. 313-06961); Lane 2. Restriction digestion of pIRES plasmid (control) using NheI-HF and EcoRI-HF. Lane 3-6. Restriction digestion of TGGAA plasmid obtained from four different clones using NheI-HF and EcoRI-HF. Inserts were shown in a white box. **(E)** Size analysis of the of TTTCA plasmids. Lane 1. Size maker, Gene Ladder Wide 1 (0.1-20 kbp) (Nippon genetics co. 313-06961); Lane 2,6. Undigested plasmids; Lane 3,7. Restriction digestion of pIRES plasmid (control) using NheI-HF and EcoRI-HF. Lane 4,5,8,9. Restriction digestion of TTTCA plasmids obtained from four different clones using NheI-HF and EcoRI-HF. Inserts were shown in white boxes. Lane 10. Size maker, Gene Ladder 100 (0.1–2 kbp) (Nippon genetics co. 316-06951).

**FIGURE 2:**
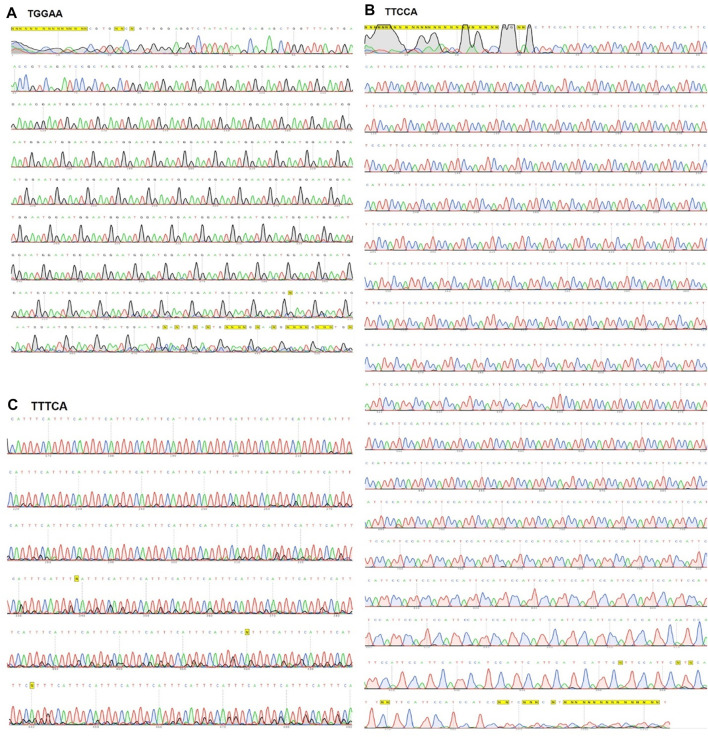
Sanger sequencing result for SCA31 and BAFME1 repeats. **(A)** TGGAA—Up to 500 bp sequence from the 5′side of a plasmid containing 2.5 kb SCA31 insert was confirmed by Sanger sequencing. **(B)** TTCCA—Up to 1 kbp sequence from the 3′side of the SCA31 plasmid was confirmed by Sanger sequencing. **(C)** TTTCA—About 180 units of the BAFME1 repeat of was inserted into the vector. Up to 400 bp sequence from the 5′side was confirmed by Sanger sequencing.

The initial oligonucleotide containing 80 nucleotides was selected for our experiment, as the circularized template exhibits maximum amplification efficiency for this size (Joffroy et al., 2018). The formation of circular DNA was confirmed using 15% acrylamide/8 M urea denaturing gel (Figure 1A; Supplementary Figure S1A, B). The size discrepancy between marker and ssDNA (Figure 1A; Supplementary Figure S1A) may be due to base composition bias of ssDNA causing differential mobility. The reaction mixture of circularization was treated with a single-strand specific nuclease, Exonuclease T. Unreacted linear ssDNA found in lane 3 of Supplementary Figure S1B was digested and not found in lane 5, which showed only circular DNA was left. Alternatively, Exonuclease I may be more effective for ssDNA containing C nucleotide(s). After removing linear ssDNA, RCA was employed for 1 h to SCA31 (repeat unit is TTGGA) and M13_90mer circular DNA (control). Ladder like pattern (Kuhn and Frank-Kamenetskii, 2005) was observed for control (M13_90mer) and smear was observed for concatenated repeat sequence showing the success of RCA (Figure 1B). In order to produce a longer repeat sequence, the RCA reaction was extended to 12 h; consequently, extremely large RCA products were obtained, which were concatemers of the repeat DNA (Figure 1C). DNA ranging from 2 to 3 kbp was cut out from the randomly elongated RCA products and cloned into pIRES vector (Figure 1D). Sanger sequence result for one clone confirmed at least 90-unit repeats of TGGAA from the 5′ end (Figure 2A) and 180-unit repeats of TTCCA from the 3’ end (Figure 2B). The same procedure was applied for the BAFME1 repeat (repeat unit is TTTCA). Figure 1E white boxes shows that inserts of about 600 bp (Lane 4-5) and about 900 bp (Lane 8-9) were cloned in the pIRES vector. We confirmed the sequence of one clone and found that the insert contained at least 80-unit repeats of TTTCA (Figure 2C). These results show the reproducibility of this method; repetitive sequences of different repeat sequences with various unit lengths could be generated by RCA and be cloned into plasmid vectors.

To characterize the whole sequence composition and repeat unit length, Oxford Nanopore Technology (ONT) was attempted on a SCA31 plasmid containing a 2.5 kbp repeat. From 8,000 reads (N50: 6,833bp), 3,831 reads were extracted with 6,000-8,000 bp length (almost full length of the plasmid), which were assembled to form the plasmid containing the repeat and vector (Supplementary Figure S2A). Supplementary Figure S2B shows 50 random reads aligned onto the consensus sequence. After polishing with the Medaka tool, full length sequence of the plasmid was obtained (Supplementary Figure S2C), containing 445 TGGAA repeats that included four mutated sequences—TGTAA, TTAAA, TGGAGG, TGGAT. Although these point mutations were found in the sequence, the longest stretch of a perfect TGGAA repeat was 1960 bp (392 TGGAA), which covered the pathogenic size. In summary, our method successfully obtained uninterrupted long repeat sequences using Bst DNA polymerase. However, our trial with phi29 polymerase was not successful despite trying with various concentrations and different reaction conditions (Supplementary Figure S3). RCA products did not migrate from the loading well into the gel and did not run after treating with various concentrations of mung bean nuclease. As a result, the DNA could not be extracted out from the gel. Our method can be used for generating long tandem repeats of tailored size in a cell-free manner and combined with the ADER method (Osborne and Thornton, 2008) to stabilize the repeat units in *E. coli*. Moreover, this *in vitro* cloning method may be applicable for other types of repeat expansion, including those for neurodegenerative diseases, undiscovered disease-causative repeats or artificial repeats and may be used to produce animal and cell culture models to study repeat expansion diseases *in vivo* and *in vitro*. Lastly, we believe that this method can also be used for studying the function of repeats in the genome, such as alpha-satellite, centromeres and telomeres.

## Data Availability

The original contributions presented in the study are included in the article/Supplementary Material, further inquiries can be directed to the corresponding author/s.
