# Supplementary material for: Synthesis and cloning of long repeat sequences using single-stranded circular DNA
Source: Front Bioeng Biotechnol. 2023 Mar 9;11:1115159. doi: 10.3389/fbioe.2023.1115159 (PMC10033958; doi:10.3389/fbioe.2023.1115159)
Supplement: Supplementary file 1 [file DataSheet2.PDF]

Table 1. Sequences for oligonucleotides used in ssDNA template preparation, rolling circle amplification, PCR amplification check and Sanger sequencing.

Oligonucleotides	Sequence (5’−3’)
80nt TGGAA ssDNA	TGGAATGGAATGGAATGGAATGGAATGGAATGGAATGGAATGGAA TGGAATGGAATGGAATGGAATGGAATGGAATGGAA
Sca31_RCA_F	TGGAATGGAATGGAA
Sca31_RCA_R	TTCCATTCCATTCCA
pIRES_F	GTAACAACTCCGCCCCATT
pIRES_R	GGTACCGTCGACTGCAGAA
M13_90mer ssDNA (Control for RCA)	GTAAAACGACGGCCAGTAAACAGTGACCATGATAGTGGCCACCCTG CAACCGTGTTGTTTGTCAGGTTCATTTGTCATAGCTGTTTCCTG
M13_RCA_F	CTGCAACCGTGTTGTTT
M13_RCA_R	GGTGGCCACTATCAT
80 nt TTTCA ssDNA	TTTCATTTCATTTCATTTCATTTCATTTCATTTCATTTCATTTCATTTC ATTTCATTTCATTTCATTTCATTTCATTTCA
BAFME1_RCA_F	TTTCATTTCATTTCA
BAFME1_RCA_R	TGAAATGAAATGAAA
