# Supplementary material for: Synthesis and cloning of long repeat sequences using single-stranded circular DNA
Source: Front Bioeng Biotechnol. 2023 Mar 9;11:1115159. doi: 10.3389/fbioe.2023.1115159 (PMC10033958; doi:10.3389/fbioe.2023.1115159)
Supplement: Supplementary file 2 [file DataSheet1.PDF]

Fig. S1

A.

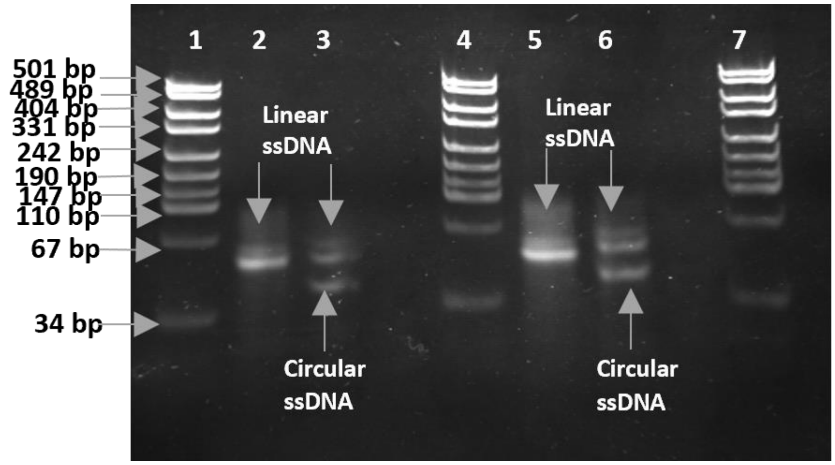

B.

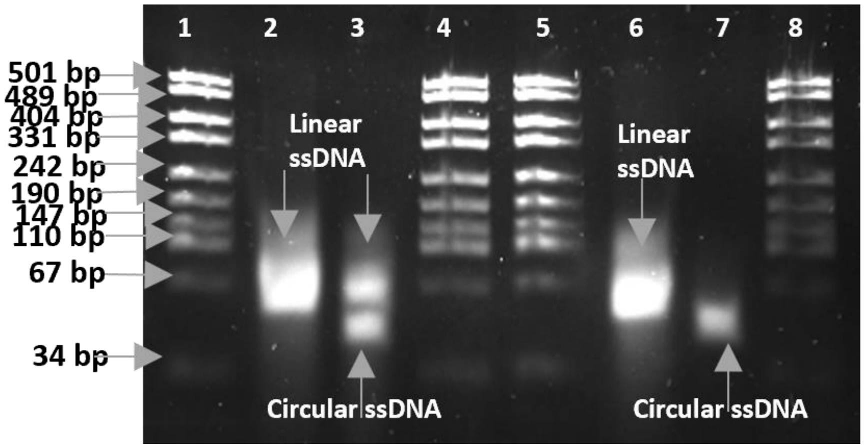

Fig. S1 A. Circular ssDNA formation (whole picture of Fig. 1) Lane 1,4,7. pUC19 DNA/MspI (HpaII) Marker (Thermo Scientific); Lane 2,5. 80 nt linear ssDNA oligos (upper bands); Lane 3,6. Unreacted 80 nt linear ssDNA oligos (upper bands), and 80 nt circular ssDNA product (lower bands). **B. Confirmation of circular ssDNA product**. Lane 1,4,5,8. pUC19 DNA/MspI (HpaII) Marker (Thermo Scientific); Lane 2,6. 80 nt linear ssDNA oligos (upper bands); Lane 3 Unreacted 80 nt linear ssDNA oligos (upper bands), and 80 nt circular ssDNA product (lower bands). Lane

7. Exonuclease treatment for the reaction mixture of lane 3 eliminated the linear ssDNA bands, leaving circular ssDNA (lower band).

Fig. S2

A.

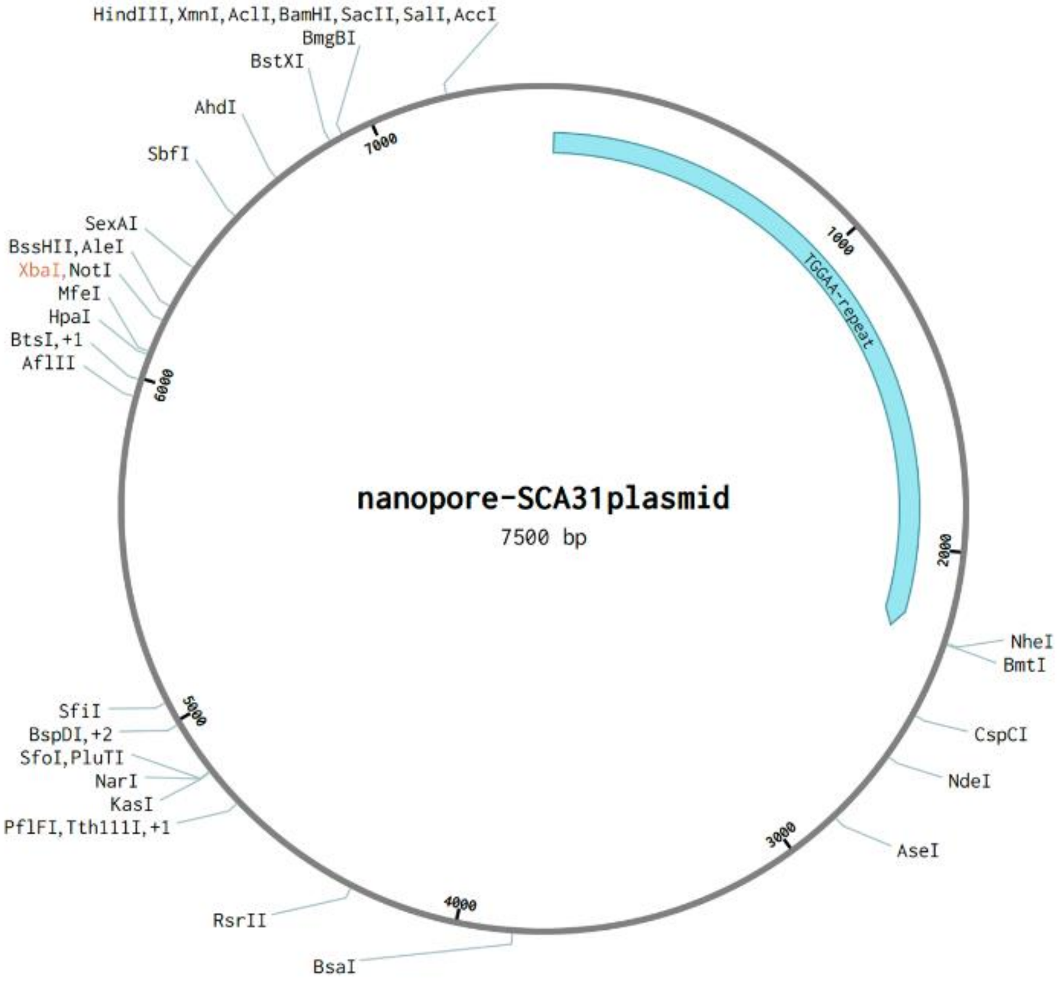

B.

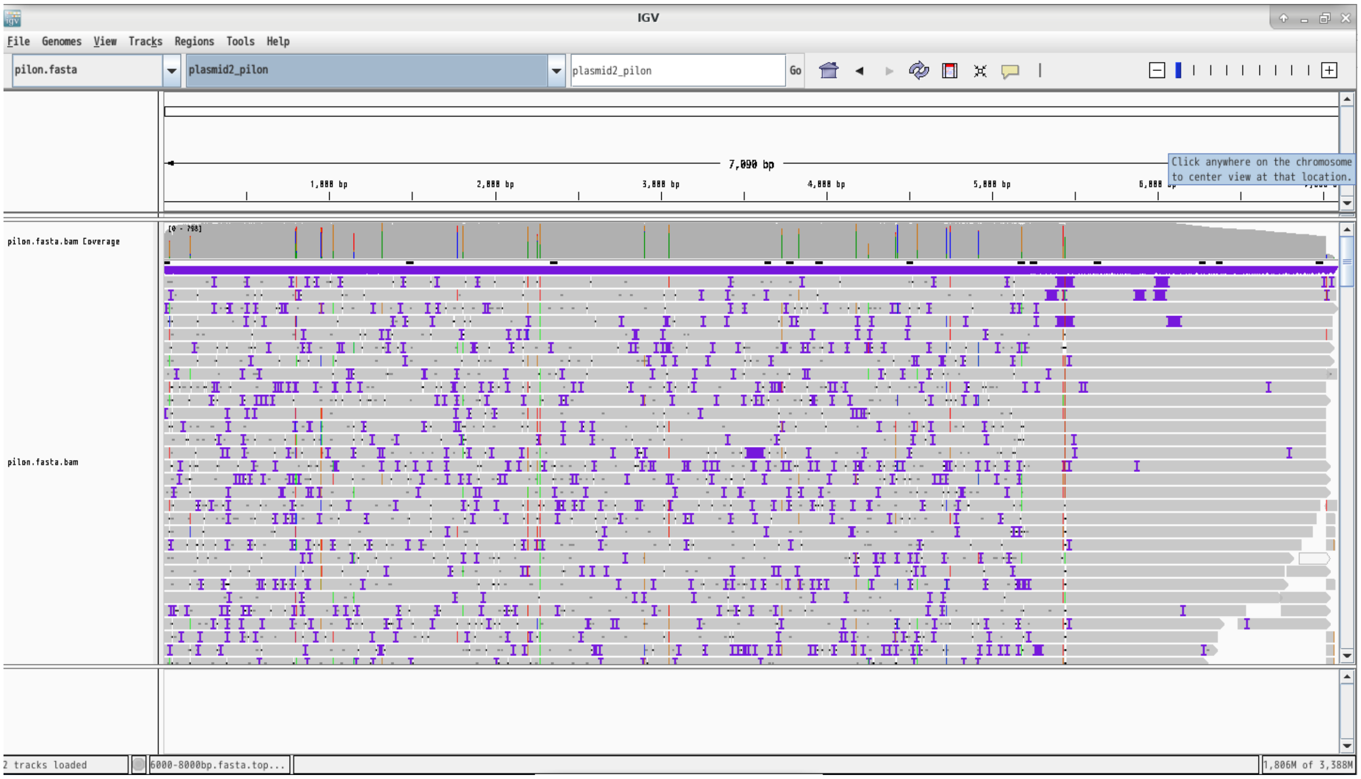

C.

GTTCCAGTTACGTATTACTAATTCGAAAAAGAATGGAATGGAATGTAATTAAATGGAATGGAATGGAATG
GAATGGAATGGAATGGAATGGAATGGAATGGAATGGAATGGAATGGAATGGAATGGAATGGAATGGAATG
GAATGGAATGGAATGGAATGGAATGGAATGGAATGGAATGGAATGGAATGGAATGGAATGGAATGGAATG
GAATGGAATGGAATGGAATGGAATGGAATGGAATGGAGGTGGAATGGAATGGAATGGAATGGAAT
GGAATGGAATGGAATGGAATGGAATGGAATGGAATGGAATGGAATGGAATGGAATGGAATGGAATGGAAT
GGAATGGAATGGAATGGAATGGAATGGAATGGAATGGAATGGAATGGAATGGAATGGAATGGAATGGAAT
GGAATGGAATGGAATGGAATGGAATGGAATGGAATGGAATGGAATGGAATGGAATGGAATGGAATGGAAT
GGAATGGAATGGAATGGAATGGAATGGAATGGAATGGAATGGAATGGAATGGAATGGAATGGAATGGAAT
GGAATGGAATGGAATGGAATGGAATGGAATGGAATGGAATGGAATGGAATGGAATGGAATGGAATGGAAT
GGAATGGAATGGAATGGAATGGAATGGAATGGAATGGAATGGAATGGAATGGAATGGAATGGAATGGAAT
GGAATGGAATGGAATGGAATGGAATGGAATGGAATGGAATGGAATGGAATGGAATGGAATGGAATGGAAT
GGAATGGAATGGAATGGAATGGAATGGAATGGAATGGAATGGAATGGAATGGAATGGAATGGAATGGAAT
GGAATGGAATGGAATGGAATGGAATGGAATGGAATGGAATGGAATGGAATGGAATGGAATGGAATGGAAT
GGAATGGAATGGAATGGAATGGAATGGAATGGAATGGAATGGAATGGAATGGAATGGAATGGAATGGAAT
GGAATGGAATGGAATGGAATGGAATGGAATGGAATGGAATGGAATGGAATGGAATGGAATGGAATGGAAT
GGAATGGAATGGAATGGAATGGAATGGAATGGAATGGAATGGAATGGAATGGAATGGAATGGAATGGAAT
GGAATGGAATGGAATGGAATGGAATGGAATGGAATGGAATGGAATGGAATGGAATGGAATGGAATGGAAT
GGAATGGAATGGAATGGAATGGAATGGAATGGAATGGAATGGAATGGAATGGAATGGAATGGAATGGAAT
GGAATGGAATGGAATGGAATGGAATGGAATGGAATGGAATGGAATGGAATGGAATGGAATGGAATGGAAT
GGAATGGAATGGAATGGAATGGAATGGAATGGAATGGAATGGAATGGAATGGAATGGAATGGAATGGAAT
GGAATGGAATGGAATGGAATGGAATGGAATGGAATGGAATGGAATGGAATGGAATGGAATGGAATGGAAT
GGAATGGAATGGAATGGAATGGAATGGAATGGAATGGAATGGAATGGAATGGAATGGAATGGAATGGAAT

GGAATGGAATGGAATGGAATGGAATGGAATGGAATGGAATGGAATGGAATGGAATGGAATGGAATGGAAT
GGAATGGAATGGAATGGAATGGAATGGAATGGAATGGAATGGAATGGAATGGAATGGAATGGAATGGAAT
GGAATGGAATGGAATGGAATGGAATGGAATGGAATGGAATGGAATGGAATGGAATGGAATGGAATGGAAT
GGAATGGAATGGAATGGAATGGAATGGAATGGAATGGAATGGAATGGAATGGAATGGAATGGAATGGAAT
GGAATGGAATGGAATGGAATGGAATGGAATGGAATGGAATGGAATGGAATGGAATGGAATGGAATGGAAT
GGAATGGAATGGAATGGAATGGAATGGAATGGAATGGAATGGAATGGAATGGAATGGAATGGAATGGAAT
GGAATGGAATGGAATGGAATGGAATGGAATGGAATGGAATGGAATGGAATGGAATGGAATGGAATGGAAT
GGAATGGAATGGAATGGAATGGAATGGAATGGAATGGAATGGAATGGAATGGAATGGAATGGAATGGAAT
GGAATGGAATGGAATGGAATGGAATGGAATGGAATGGAATGGAATGGAATGGAATGGAATGGAATGGAAT
GGAATGGAATGGAATGGAATGGAATGGAATGGAATGGAATGGAATGGATTGGAATGGAATGGAATGGAAT
GGAATGGAATGGAATGGAAGCTAGCGGATCTGACGGTTCACTAAACCAGCTCTGCTTATATAGACCTCCC
ACCGTACACGCCTACCGCCCATTTGCGTCAATGGGGCGGAGTTGTTACGACATTTTGGAAAGTCCCGTTG
ATTTTGGTGCCAAAACAAACTCCCATTGACGTCAATGGGGTGGAGACTTGGAAATCCCCGTGAGTCAAAC
CGCTATCCACGCCCATTGATGTACTGCCAAAACCGCATCACCATGGTAATAGCGATGACTAATACGTAGA
TGTACTGCCAAGTAGGAAAGTCCCATAAGGTCATGTACTGGGCATAATGCCAGGCGGGCCATTTACCGTC
ATTGACGTCAATAGGGGGCGTACTTGGCATATGATACACTTGATGTACTGCCAAGTGGGCAGTTTACCGT
AAATACTCCACCCATTGACGTCAATGGAAAGTCCCTATTGGCGTTACTATGGGAACATACGTCATTATTG
ACGTCAATGGGCGGGGGTCGTTGGGCGGTCAGCCAGGCGGGCCATTTACCGTAAGTTATGTAACGCGGAA
CTCCATATATGGGCTATGAACTAATGACCCCGTAATTGATTACTATTAATAACTAATGCATGGCGGTAAT
ACGGTTATCCACAGAATCAGGGGATAACGCAGGAAAGAACATGTGAGCAAAAGGCCAGCAAAAGGCCAGG
AACCGTAAAAAGGCCGCGTTGCTGGCGTTTTTCCATAGGCTCCGCCCCCCTGACGAGCATCACAAAAATC
GACGCTCAAGTCAGAGGTGGCGAAACCCGACAGGACTATAAAGATACCAGGCGTTTCCCCCTGGAAGCTC
CCTCGTGCGCTCTCCTGTTCCGACCCTGCCGCTTACCGGATACCTGTCCGCCTTTCTCCCTTCGGGAAGC
GTGGCGCTTTCTCATAGCTCACGCTGTAGGTATCTCAGTTCGGTGTAGGTCGTTCGCTCCAAGCTGGGCT
GTGTGCACGAACCCCCCGTTCAGCCCGACCGCTGCGCCTTATCCGGTAACTATCGTCTTGAGTCCAACCC
GGTAAGACACGACTTATCGCCACTGGCAGCAGCCACTGGTAACAGGATTAGCAGAGCGAGGTATGTAGGC
GGTGCTACAGAGTTCTTGAAGTGGTGGCCTAACTACGGCTACACTAGAAGAACAGTATTTGGTATCTGCG
CTCTGCTGAAGCCAGTTACCTTCGGAAAAAGAGTTGGTAGCTCTTGATCCGGCAAACAAACCACCGCTGG
TAGCGGTGGTTTTTTTGTTTGCAAGCAGCAGATTACGCGCAGAAAAAAAGGATCTCAAGAAGATCCTTTG
ATCTTTTCTACGGGGTCTGACGCTCAGTGGAACGAAAACTCACGTTAAGGGATTTTGGTCATGAGATTAT
CAAAAAGGATCTTCACCTAGATCCTTTTAAATTAAAAATGAAGTTTTAAATCAATCTAAAGTATATATGA
GTAACCTGAGGCTATGGCAGGGCCTGCCGCCCCGACGTTGGCTGCGAGCCCTGGGCCTTCACCCGAACTT
GGGGGGTGGGGTGGGGAAAAGGAAGAAACGCGGGCGTATTGGCCCCAATGGGGTCTCGGTGGGGTATCGA
CAGAGTGCCAGCCCTGGGACCGAACCCCGCGTTTATGAACAAACGACCCAACACCGTGCGTTTTATTCTG
TCTTTTTATTGCCGTCATAGCGCGGGTTCCTTCCGGTATTGTCTCCTTCCGTGTTTCAGTTAGCCTCCCC
CTAGGGTGGGCGAAGAACTCCAGCATGAGATCCCCGCGCTGGAGGATCATCCAGCCGGCGTCCCGGAAAA
CGATTCCGAAGCCCAACCTTTCATAGAAGGCGGCGGTGGAATCGAAATCTCGTGATGGCAGGTTGGGCGT
CGCTTGGTCGGTCATTTCGAACCCCAGAGTCCCGCTCAGAAGAACTCGTCAAGAAGGCGATAGAAGGCGA
TGCGCTGCGAATCGGGAGCGGCGATACCGTAAAGCACGAGGAAGCGGTCAGCCCATTCGCCGCCAAGCTC
TTCAGCAATATCACGGGTAGCCAACGCTATGTCCTGATAGCGGTCCGCCACACCCAGCCGGCCACAGTCG
ATGAATCCAGAAAAGCGGCCATTTTCCACCATGATATTCGGCAAGCAGGCATCGCCATGGGTCACGACGA
GATCCTCGCCGTCGGGCATGCTCGCCTTGAGCCTGGCGAACAGTTCGGCTGGCGCGAGCCCCTGATGCTC
TTCGTCCAGATCATCCTGATCGACAAGACCGGCTTCCATCCGAGTACGTGCTCGCTCGATGCGATGTTTC
GCTTGGTGGTCGAATGGGCAGGTAGCCGGATCAAGCGTATGCAGCCGCCGCATTGCATCAGCCATGATGG
ATACTTTCTCGGCAGGAGCAAGGTGAGATGACAGGAGATCCTGCCCCGGCACTTCGCCCAATAGCAGCCA
GTCCCTTCCCGCTTCAGTGACAACGTCGAGCACAGCTGCGCAAGGAACGCCCGTCGTGGCCAGCCACGAT
AGCCGCGCTGCCTCGTCTTGCAGTTCATTCAGGGCACCGGACAGGTCGGTCTTGACAAAAAGAACCGGGC
GCCCCTGCGCTGACAGCCGGAACACGGCGGCATCAGAGCAGCCGATTGTCTGTTGTGCCCAGTCATAGCC
GAATAGCCTCTCCACCCAAGCGGCCGGAGAACCTGCGTGCAATCCATCTTGTTCAATCATGCGAAACGAT
CCTCATCCTGTCTCTTGATCGATCTTTGCAAAAGCCTAGGCCTCCAAAAAAGCCTCCTCACTACTTCTGG
AATAGCTCAGAGGCCGAGGCGGCCTCGGCCTCTGCATAAATAAAAAAAATTAGTCAGCCATGGGGCGGAG
AATGGGCGGAACTGGGCGGAGTTAGGGGCGGGATGGGCGGAGTTAGGGGCGGGACTATGGTTGCTGACTA
ATTGAGATGCATGCTTTGCATACTTCTGCCTGCTGGGGAGCCCGGGGACTTTCCACACCCGGTTGCTGAC
TAATTGAGATGCATGCTTTGCATACTTCTGCCTGCTGGGGAGCCCGGGGACTTTCCACACCCTAACTGAC

ACACATTCCACAGCTGGTTCTTTCCGCCTCAGGACTCTTCCTTTTTCAATATTATTGAAGCATTTATCAG
GGTTATTGTCTCATGAGCGGATACATATTTGAATGTATTTAGAAAAATAAACAAATAGGGGTTCCGCGCA
CATTTCCCCGAAAAGTGCCACCTGACGCGCCCTGTAGCGGCGCATTAAGCGCGGCGGGTGTGGTGGTTAC
GCGCAGCGTGACCGCTACACTTGCCAGCGCCCTAGCGCCCGCTCCTTTCGCTTTCTTCCCTTCCTTTCTC
GCCACGTTCGCCGGCTTTCCCCGTCAAGCTCTAAATCGGGGGCTCCCTTTAGGGTTCCGATTTAGTGCTT
TACGCCACCTCGACCCCAAAAAACTTGATTAGGGTGATGGTTCACGTAGTGGGCCATCGCCCTGATAGAC
GGTTTTTCGCCCTTTGACGTTGGAGTCCACGTTCTTTAATAGTGGACTCTTGTTCCAAACTGGAACAACA
CTCAACCCTATCTCGGTCTATTCTTTTGATTTATAAGGGATTTTGCCGATTTCGGCCTATTGGTTAAAAA
ATGAGCTGATTTAACAAAAATTTAACGCGAATTTTAACAAAATATTAACGCTTACAATTTACGCCTTAAG
ATACATTGATGAGTTTGGACAAACCACAACTAGAATGCAGTGAAAAAAATGCTTTATTTGTGAAATTTGT
GATGCTATTGCTTTATTTGTAACCATTATAAGCTGCAATAAACAAGTTAACAACAACAATTGCATTCATT
TTATGTTTCAGGTTCAGGGGGAGGTGTGGGAGGTTTTTTAAAGCAAGTAAAACCTCTACAAATGTGGTAT
GGCTGATTATGATCTAGAGTCGCGGCCGCCTACTGGAACAGGTGGTGGCGGGCCTCGGCGCGCTCGTACT
GCTCCACCACGGTGTAGTCCTCGTTGTGGGAGGTGATGTCCAGCTTGGAGTCCACGTAGTAGTAGCCGGG
CAGCTTCACGGGCTTCTTGGCCATGTAGATTGACTTGAACTCCACCAGGTAGTGGCCGCCGCCCTTCAGC
TTCAGCGCCTTGTGGATCTCGCCCTTCAGCACGCCGTCGCGGGGGTACAGGCGCTCGGTGGAGGGCTCCC
AGCCCAGAGTCTTCTTCTGCATTACGGGGCCGTCGGAGGGGAAGTTCACGCCGATGAACTTCACGTGGTA
GATGAAGGTGCCGTCCTGCAGGGAGGAGTCCCGGGTCACGGTCACCACGCCGCCGTCCTCGAAGTTCATC
ACGCGCTCCCACTTGAAGCCCTCGGGGAAGGACAGCTTCTTGTAGTCGGGGATGTCGGCGGGGTGCTTCA
CGTACACCTTGGAGCCGTACTGGAACTGGGGGGACAGGATGTCCCGGGCGAAGGGCAGGGGGCCGCCCTT
GGTCACCTGCAGCTTGGCGGTCTGGGTGCCCTCGTAGGGCTTGCCCTCGCCCTCGCCCTCGATCTCGAAC
TCGTGGCCGTTCACGGAGCCCTCCATGTGCACCTTGAAGCGCATGAAGGGCTTGATGACGTTCTCAGTGC
TATCCATGGTTGTGGCCATATTATCATCGTGTTTTTCAAAGGAAAACCACGTCCCCGTGGTTCGGGGGGC
CTAGACGTTTTTTTAACCTCGACTAAACACATGTAAAGCATGTGTACCGAGGCCCCAGATCAGATCCCAT
ACAATGGGGTACCTTCTGGGCATCCTTCAGCCCCTTGTTGAATACGCTTGAGGAGAGCCATTTGACTCTT
TCCACAACTATCCAACTCACAACGTGGCACTGGGGTTGTGCCGCCTTTGCAGGTGTATCTTATACACGTG
GCTTTTGGCCGCAGAGGCACCTGTCGCCAGGTGGGGGGTTCCGCTGCCTGCAAAGGGTCGCTACAGACGT
TGTTTGTCTTCAAGAAGCTTCCAGAGGAACTGCTTCCTTCACGACATTCAACAGACCTTGCATTCCTTTG
GCGAGAGGGGAAAGACCCCTAGGAATGCTCGTCAAGAAGACAGGGCCAGGTTTCCGGGCCCTCACATTGC
CAAAAGACGGCAATATGGTGGAAAATAACATATAGACAAACGCACACCGGCCTTATTCCAAGCGGCTTCG
GCCAGTAACGTTAGGGGGGGGGAGGGAGAGGGGCGGATCCCGGGCCCGCGGTACCGTCGACTGCAGAATTA
GCAATACGTA

Fig. S2 A. Physical map constructed from nanopore sequencing of SCA31 plasmid containing 2.5 kbp TGGAA repeats. **B.** 50 raw nanopore reads were mapped, aligned, and visualized with Integrative Genomics Viewer (IGV). **C.** SCA31 plasmid sequenced by ONT.

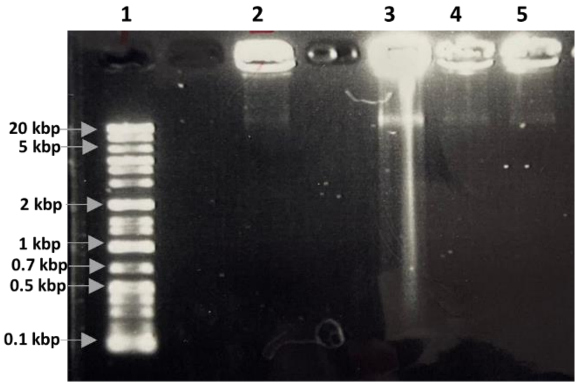

Fig. S3 RCA products by phi29 DNA polymerase. Lane 1. Size maker, Gene Ladder Wide 1 (0.1-20 kbp) (Nippon genetics co. 313-06961); Lane 2. RCA for SCA31 (12 h); Lane 3. RCA product digested with 10 U Mung bean nuclease. Lane 4. RCA product digested with 1 U Mung bean nuclease. Lane 5. RCA product digested with 0.1 U Mung bean nuclease.
